# A randomised controlled trial testing a web-based, computer-tailored self-management intervention for people with or at risk for chronic obstructive pulmonary disease: a study protocol

**DOI:** 10.1186/1471-2458-13-557

**Published:** 2013-06-07

**Authors:** Viola Voncken-Brewster, Huibert Tange, Hein de Vries, Zsolt Nagykaldi, Bjorn Winkens, Trudy van der Weijden

**Affiliations:** 1 School for Public Health and Primary Care (CAPHRI), Department of General Practice, Maastricht University Medical Centre, PO Box 616, 6200MD, Maastricht, Netherlands; 2 School for Public Health and Primary Care (CAPHRI), Department of Health Promotion, Maastricht University Medical Centre, Maastricht, Netherlands; 3 Health Sciences Centre, Department of Family and Preventive Medicine, University of Oklahoma, Oklahoma City, OK, USA; 4 School for Public Health and Primary Care (CAPHRI), Department of Methodology and Statistics, Maastricht University Medical Centre, Maastricht, Netherlands

**Keywords:** Internet intervention, Tailoring, Randomised controlled trial, Chronic obstructive pulmonary disease, Self-management, Health behaviour, Chronic disease

## Abstract

**Background:**

Chronic Obstructive Pulmonary Disease (COPD) is a major cause of morbidity and mortality. Effective self-management support interventions are needed to improve the health and functional status of people with COPD or at risk for COPD. Computer-tailored technology could be an effective way to provide this support.

**Methods/Design:**

This paper presents the protocol of a randomised controlled trial testing the effectiveness of a web-based, computer-tailored self-management intervention to change health behaviours of people with or at risk for COPD. An intervention group will be compared to a usual care control group, in which the intervention group will receive a web-based, computer-tailored self-management intervention. Participants will be recruited from an online panel and through general practices. Outcomes will be measured at baseline and at 6 months. The primary outcomes will be smoking behaviour, measuring the 7-day point prevalence abstinence and physical activity, measured in minutes. Secondary outcomes will include dyspnoea score, quality of life, stages of change, intention to change behaviour and alternative smoking behaviour measures, including current smoking behaviour, 24-hour point prevalence abstinence, prolonged abstinence, continued abstinence and number of quit attempts.

**Discussion:**

To the best of our knowledge, this will be the first randomised controlled trial to test the effectiveness of a web-based, computer-tailored self-management intervention for people with or at risk for COPD. The results will be important to explore the possible benefits of computer-tailored interventions for the self-management of people with or at risk for COPD and potentially other chronic health conditions.

**Dutch trial register:**

NTR3421

## Background

Chronic Obstructive Pulmonary Disease (COPD) is a leading cause of morbidity and mortality, causing a major public health problem [1]. The disease is characterized by a progressive decline in lung function, leading to a deteriorating health status and quality of life [1]. COPD is not curable, but self-management and behaviour modification can delay further decline of health status [2].

The growing burden of COPD highlights the need for effective self-management interventions. However, only a limited number of studies have examined the effectiveness of self-management interventions and mostly in small samples [3]. These self-management interventions tend to focus on patient education. However, there is a trend to broaden this approach through personal counselling with a health care provider in order to give patients information that is relevant to their circumstances [3]. Another trend in health promotion interventions includes the use of information and communication technology, also referred to as e-Health [4].

E-Health interventions have expanded immensely during the past decade focusing on disease management and prevention [4]. Multiple e-Health interventions have been developed for COPD patients [5-7]. E-Health interventions can provide patients with health promotion information available at any time, in their own home [8]. To individualize e-Health interventions, developers may use computer-tailored technology. A computer-tailored e-Health application provides personally relevant feedback, by tailoring information to the individual needs and characteristics of the person [9], which results in increased attention, appreciation, and processing of information [10,11]. Over the years multiple variations of tailoring programs have been developed and this intervention technique has shown its potential to effectively support health behaviour change [12-14], for example smoking cessation, physical activity and interventions targeting multiple behaviours [11,15,16]. Computer-tailoring has the ability to reach large audiences at a relatively low cost [17] and tailored health promotion information is found to be more cost-effective compared to usual care [18] and motivational interviewing [19]. However, to our knowledge this technique has not been used to promote self-management of COPD patients.

In the MasterYourBreath (AdemDeBaas in Dutch) project we developed a computer-tailored self-management intervention for COPD patients. We evaluated the usability of the prototype [20] and conducted a pilot study to assess the feasibility of integrating the self-management intervention into primary care. The goal of the current study is to test the effectiveness of a web-based, computer-tailored self-management intervention for people at risk for or with COPD. This paper describes the study protocol.

## Methods

### Study design

This is a two-arm randomised controlled trial, comparing a web-based, computer-tailored self-management intervention with usual care. The study contains a design with a baseline measurement and a follow-up measurement after 6 months. We hypothesise that participants receiving a web-based, computer-tailored self-management intervention will achieve higher smoking cessation rates and obtain higher levels of physical activity compared to the control group. Moreover, we hypothesise that the intervention group will move along the stages of change continuum, have an increased intention to change their behaviour, decrease their level of disability and obtain a better COPD-related quality of life, compared to the control group. We will carry out a process evaluation in addition to this effect evaluation. The study was approved by the Medical Ethical Committee of Maastricht University Medical Centre (METC 12-4-033).

### Effect evaluation

#### Recruitment

Adults between 40 and 70 years of age, who are proficient in Dutch, have access to a computer with internet access, have basic computer skills and are diagnosed with COPD or are at moderate or high risk for COPD, are eligible for participation. The Respiratory Health Screening Questionnaire (RHSQ) [21] is used to determine whether a person has a moderate or high risk for COPD. Participants are recruited using two methods, i.e. from an existing Dutch online panel assembled by the company Flycatcher (http://www.flycatcher.eu) and from 5 general practices. Flycatcher is an institute for online research certified by the International Organization for Standardization (ISO). The total online panel is a representative sample of Dutch internet users. All active members of the online panel between 40 and 70 years of age will be invited. In case of more than one person per home address, one individual will be randomly selected and invited. Subsequently, members complete the RHSQ questionnaire to be screened for eligibility. Based on Dutch literature, we estimate that 31% of the people between 40 and 70 years old have COPD or a moderate or high risk of having COPD [22]. Members of the online panel receive a small incentive after participating in a certain number of research projects. The 5 general practices were recruited for another ongoing project in which patients are screened for COPD by their general practitioner using the RHSQ. This project examines the implementation of the RHSQ in primary care and does not aim to improve the self-management of participants. Eligible patients will be sent an invitation letter by mail for the MasterYourBreath study. All participants receive an online study information letter and have to complete an online informed consent form by checking a check box, before entering the study.

#### Sample size calculation

The sample size for smoking is based on the assumption that 49.2% of the population with an increased COPD risk smokes [22]. A sample size calculation by PS software [23] indicates that 446 participants will be necessary in each arm at the end of the trial to detect 20% 7-day point prevalence abstinence in the intervention group, compared to 10% in the control group [24], with a 80% power and a significance level of 0.05. Since we do not uphold an upper bound for minutes a person can be physically active, 100% of the participants will be included in the analyses for physical activity. In another study the standard deviation for the Dutch population was 26.63 [25]. Considering this, we will need 112 participants in each arm, which is feasible to obtain from the suggested sample of 446 participants in order to detect a difference of 10 minutes with 80% power and a 0.05 significance level.

#### Randomisation

**Figure 1:**
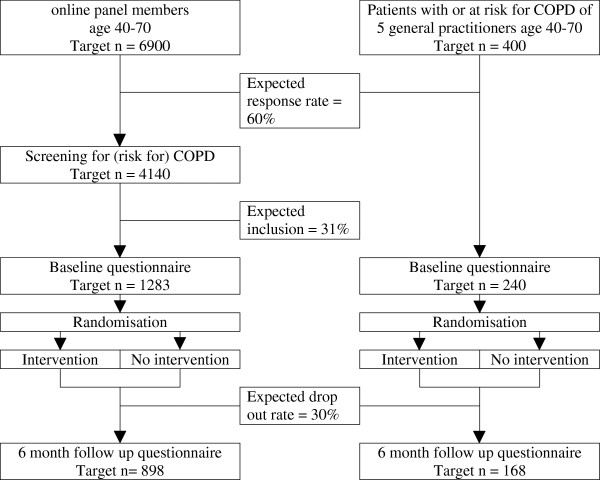
Overview of the recruitment and randomisation of participants.

Participants will be randomly allocated to intervention and control groups after filling out the baseline questionnaire. Randomization will be stratified by method of recruitment (online or through general practice) and participants are randomised within each stratum using a permuted block design with a random block size varying from 4 to 20. This approach helps us achieve balanced and evenly distributed samples in both groups following different recruitment strategies. A researcher who is not directly involved in other aspects of the study will perform the randomisation using PROC PLAN in Statistical Analysis System v9.1 software (SAS Institute, Cary, NC). Figure 1 shows an overview of the recruitment and randomisation process.

### Web-based intervention

#### Framework

The web-based intervention in this project was created with ‘Tailorbuilder’ software package (OverNite Software Europe B.V., The Netherlands), an internet application based on Perl and a MySQL5 database, which includes web-based questionnaires to identify user characteristics, a database with feedback messages and an algorithm with routing procedures and tailoring rules (algorithm). Feedback messages are derived from participants’ answers. Responses are linked to relevant messages [9]. Message selection and linking occurs by means of routing procedures and tailoring rules using principles of previously designed computer-tailored studies [11,26]. Messages are displayed after participants have filled out the questionnaire. The web-based intervention consists of two behaviour change modules, including smoking cessation and physical activity.

#### Behaviour change modules

The web-based intervention is based on modules developed in earlier studies targeting smoking cessation and physical activity for the general population [11,27]. We adapted these modules for COPD patients and for people at risk for COPD. We used the I-Change model as theoretical framework in our intervention [28]. This model includes several health promotion theories, i.e.: the Attitude-Social influence-Self-efficacy model (ASE) [29], which can be thought of as an incorporation of ideas of the Theory of Planned Behavior [30], the Social Cognitive Theory [31], the Transtheoretical Model [32], the Health Belief Model [33], and Implementation and Goal setting theories [31,34,35]. We evaluated and improved the usability of the behaviour change modules in a previous study [20].

The smoking cessation and physical activity modules provide participants with tailored feedback messages. Tailoring algorithms consider demographical characteristics and psychosocial constructs to generate personalized messages. The questionnaires have been tested experimentally among Dutch adults in previous studies [16,36,37]. The messages consist of texts, graphs and illustrations. Smoking cessation and physical activity modules include each the 6 intervention components described below.

1) The health risk appraisal

The health risk appraisal measures the behaviour (smoking and physical activity) and provides feedback based on Dutch guidelines for health behaviour. The feedback contains an explanation of how participants’ behaviour compares to the level of compliance with the Dutch guidelines. This is illustrated with a traffic light system, where a green light refers to meeting recommendations, a yellow light to partly meeting the recommendations and a red traffic light indicating lack of compliance with the recommendations [38]. The behavioural feedback can be tracked each time participants complete the health risk appraisal and ipsative feedback [39] will be offered regarding changes in behaviour over time. At last, a graph that shows specific behaviour changes over time is presented.

2) Motivational beliefs

The attitude toward a relevant behaviour is assessed and the perceived positive (e.g. health benefit) and negative consequences (e.g. weight gain for non-smoking) of the behaviour are addressed to confirm, correct or place participants’ beliefs in perspective.

3) Social influence

Social influences of participants’ partner, family, friends and co-workers on the specific behaviour are assessed. An explanation about how these influence the behaviour of the participants and how the participants influence others is provided. Information about how to cope with social pressure, the importance of social support and being or noticing a good role model is emphasized.

4) Goal setting and action plans

Participants can set a goal for behaviour change. They will be guided through questions to formulate the goal (e.g. starting next week I want to be physically active for 20 minutes a day, 3 times a week). Hereafter, they can choose from a list of action plans to achieve their goal and receive feedback with additional advice on how to increase the likelihood for their plans to succeed. One week after the goal is due, participants will receive an e-mail prompt to revisit this intervention component and indicate whether they achieved their goal, receive feedback and have the opportunity to adjust their goal and make new action plans.

5) Self efficacy

Barriers to perform the healthy behaviour are assessed, by asking participants which situations they find difficult. Moreover, participants are asked to indicate whether they have made plans to overcome these difficult situations (coping plans). Consequently, participants receive a list of the situations that they indicated to be difficult and are encouraged to follow through with their plans. Suggestions for plans are given if they had not already made a plan.

6) Maintenance

This component is similar to self-efficacy, but focuses on maintaining instead of changing the behaviour.

#### Levels of Tailoring

The behaviour change modules are tailored based on three levels. First, the intervention components are based on users’ preferences [10]. Participants can choose to enter one or more components per session. Second, the feedback messages are tailored to participants’ characteristics – gender, age, COPD status and level of disability. Participants’ names are used to personalize messages. Third, the feedback messages are tailored to key behaviour determinants based on psychosocial constructs. Examples of key behaviour determinants are: barriers to quitting smoking and plans to overcome them, perceived social support to be physically active, pros and cons of smoking cessation specified by participants and action plans participants make to start being more physically active.

#### Website

The behaviour change modules are embedded in a website, which can be accessed through a personal account. To keep the length of feedback messages as short as possible, the messages refer to background information and tools available via the website. The website contains background information about the MasterYourBreath project, COPD, risk for COPD, smoking and physical activity using written information, illustrations, videos and hyperlinks to other informative websites. In addition, videos of exercises that can be done at home are also included. New content will be added to the website once a month. This includes an interview with a COPD patient about coping with smoking and physical activity in her daily life and stories based on behavioural journalism to prompt participants to visit the behaviour change modules. Behavioural journalism promotes behaviour change by presenting peer modelling for cognitive processes that result in behaviour change [40]. Problems and reasons for participants choosing not to use the intervention that are communicated to the research team are leveraged to create the behavioural journalism stories in order to encourage the use of the behaviour change modules.

#### Prompts to promote technology use

The web-based intervention can be used ad libitum. Prompts will be sent to the experimental group to boost intervention use [41] with a two week time interval and some referring to new content on the website, since this may increase the number of follow-up visits [42].

If participants do not use at least one of the behaviour change modules within two weeks, they receive a prompt by e-mail to login to the website and visit a behaviour change module. Another prompt is sent out two weeks after the first prompt if participants fail to visit a behaviour change module. If participants use a behaviour change module, they receive a prompt to use the intervention again every month. This means that if participants visit both behaviour change modules, they receive a prompt every two weeks. Prompts are tailored for COPD or people at risk for COPD and refer to intervention components, new stories on the website and possible current problems with behaviour change, such as physical activity during the winter season. Participants can click on a personalised link embedded in the e-mail to access the website without having to log in.

### Data collection

A self-administered web-based questionnaire will be deployed at baseline and after 6 months. All outcome measures will be collected through this questionnaire, except for the demographic characteristics in the group that is recruited online. Flycatcher members enter their demographic variables when they start their membership. These variables are updated annually.

#### Demographic characteristics

Demographic variables include: gender, age, height, weight, country of birth, mother’s country of birth, father’s country of birth, marital status (single; in a relationship/living together; married; divorced; widowed), living situation (with parents or caregivers; alone; with partner without child(ren); with partner and child(ren); without partner with child(ren); with other people), housing (property is bought by one of the household members; property is rented by one of the household members), education level (recoded into three categories: ‘low’ (1) = primary school/basic vocational school; ‘medium’ (2) = secondary vocational school/high school degree and ‘high’(3) = higher professional degree/university degree), current work status (employed by the government; employed, but not by the government; temporary worker; self-employed; not employed), number of work hours a week (36 or more/20 to 35/12 to 19/less than 12), function (management function/non-management function), number of people managing directly and indirectly, and household income (minimum, less than 11.000 euro’s a year; less than average, between 11.000 and 23.000 euro’s a year; average, between 23.000 and 34.000 a year; 1–2 times average 34.000 to 56.000 euro’s a year; 2 times average or more 56.000 euro’s or more a year; do not know/do not want to tell).

#### Smoking

Participants are asked if they have ever smoked. Current smoking behaviour is measured by asking participants if they smoke, what they smoke (cigarettes, rolling tobacco, cigars or pipe tobacco) and how much they smoke. Questions assessing 24-hour and 7-day point prevalence abstinence (i.e., “Did you smoke during the past 24 hours, even if it was just 1 puff?” Yes; No “Did you smoke the past 7 days, even if it was just 1 puff?” Yes; No) are incorporated. To assess continued and prolonged abstinence [43] we first ask when the participants’ last serious quit date was and then measure continued abstinence (“Have you smoked since this quit date?” No, not a puff; Yes, 1–5 cigarettes or other tobacco products; Yes, more than 5 cigarettes or other tobacco products) and prolonged abstinence, allowing a grace period in which smoking behaviour will not be counted as such (“Have you smoked since two weeks after your quit date?” No, not a puff; Yes, 1–5 cigarettes or other tobacco products; Yes, more than 5 cigarettes or other tobacco products). Moreover, a question assessing the number of quit attempts, a question measuring which type of smoking cessation aids are used and the 6 item version of the Fagerström Test for Nicotine Dependence [44] are included.

#### Physical activity

Level of physical activity will be measured by the short version of the International Physical Activity Questionnaire (IPAQ) [45].

#### Health status

Participants will be asked if they are diagnosed with COPD, when they were diagnosed and if they suffer of any other chronic disease. The severity of COPD will be measured according to the standards defined by the Global Initiative for Chronic Obstructive Lung Disease (GOLD) [46]. Furthermore, the Medical research council (MRC) Dyspnoea score [47] will be administered to measure the level of disability [48].

#### Quality of life

COPD related quality of life will be measured by the Clinical COPD Questionnaire (CCQ) [49].

#### Stages of change

The stage of change [50] will be assessed for smoking (“Did you quit smoking?” Yes, I quit more than 6 months ago; Yes, I quit within the last 6 months; No, but I plan to quit within the next 30 days; No, but I plan to quit within the next 3 months; No, but I plan to quit within the next 6 months; No, and I do NOT plan to quit within the next 6 months). And physical activity (“Are you engaged in medium and/or intense physical activity for at least 30 minutes a day, 5 days a week?” Yes, since 6 months or longer; Yes, since less than 6 months; No, but I plan to be within the next 30 days; No, but I plan to be within the next 3 months; No, but I plan to be within the next 6 months; No, and I do NOT plan to be within the next 6 months).

#### Intention to change behaviour

The intention to change smoking behaviour will be measured on a 7 item likert scale (I certainly plan to quit smoking – I certainly do not plan to quit smoking) and another question measures the intention to change physical activity (I certainly plan to be more physically active – I certainly do not plan to be more physically active).

### Primary and secondary outcomes

One primary outcome measure per module is defined, including the 7-day point prevalence abstinence measuring smoking cessation and the short version of the IPAQ measuring the level of physical activity. Secondary outcomes are alternative smoking behaviour outcomes (i.e., current smoking behavior, 24-hour point prevalence abstinence, prolonged abstinence, continued abstinence and number of quit attempts), dyspnoea score, COPD related quality of life, stages of change and intention to change behaviour.

### Process evaluation

The process evaluation will be carried out in line with a framework provided by Saunders et al. [51] to examine the implementation of the MasterYourBreath study protocol and the relationship between specific intervention components and outcomes. The implementation of the study protocol will be monitored and documented by the research team. Quantitative data will be collected for program use, for example login frequency and frequency of use of each intervention component. The web-based questionnaire, will be used to ask participants of the intervention group, who did not use the intervention, to provide a reason for this (not enough time; not necessary, because I live healthy; not necessary, because I think I am not at risk for or do not have COPD; I wanted to visit the website, but I could not login to the website; other reason). Participants who used the intervention will be asked questions largely based on earlier work of de Vries et al. [11] about the website’s navigation (1 item; i.e., “It was easy to find information on the website” totally agree – totally disagree) the level of personalisation, novelty, comprehensibility and usefulness of the feedback messages (4 items; e.g., “the feedback messages were personally relevant to me” totally agree – totally disagree) and two general questions (i.e., “I would recommend MasterYourBreath to others” totally agree – totally disagree “I would like to use MasterYourBreath in the future” totally agree – totally disagree). Finally, participants will be asked to rate the feedback messages on a scale from 1 – 10. Semi-structured interviews will be conducted with participants of the experimental group to explore their reasons for participation, expectations of the study, experiences with the intervention and e-mail prompts, the recruitment procedure and information regarding the project. The interviews will be conducted after participants finish the follow-up questionnaire at 6 months, in order to avoid the contamination of the measurement. To create a heterogeneous sample, participants will be selected based on recruitment strategy (online or general practice), age, gender, COPD patient/at risk for COPD, education level, current work status and smoking status. A researcher not involved in other aspects of the study will conduct, audiotape and transcribe the interviews verbatim.

Besides, all communication between participants and the research team will be documented.

### Analysis

Number (%) and mean (SD) will be computed for categorical and numerical baseline characteristics, respectively, where the differences in these characteristics are assessed between intervention and control groups by chi-square or Fisher’s exact tests for categorical variables and independent-samples t-tests for numerical variables. Data will be analysed according to the intention to treat principle. In addition, a per-protocol analysis will be conducted. For this analysis, it is required that participants complete at least one intervention component. The uncorrected and corrected effect of the intervention on the primary and secondary outcome measures at 6 months will be assessed with logistic regression for categorical outcomes and linear regression for numerical outcomes. As for correction, baseline characteristics related to the outcome at 6 months are included in the regression model to increase the precision of the intervention effect. Since randomisation is stratified by recruitment method (online or through general practices), recruitment method is also included as a covariate in these analyses. As a sensitivity analysis, the differences in baseline characteristics between the groups recruited online and through the practices are compared. If this analysis shows that the groups are comparable, the analyses will be applied to the whole dataset, otherwise the groups will be analysed separately.

Quantitative process evaluation data will be analysed using descriptive statistics, such as number (%) and mean (SD). Differences in intervention use and satisfaction with the intervention between specific subgroups, such as gender, smoking/non-smoking and age groups, will be analysed. Content analysis will be performed to analyse the qualitative process evaluation data using the constant comparative method [52]. Statistical analysis will be performed using Statistical Package for Social Sciences (SPSS) version 19.

## Discussion

This paper presents the design of a randomised controlled trial, testing the effectiveness of a computer-tailored intervention on the self-management of people with or at risk for COPD. To our knowledge, only limited research has been conducted to evaluate self-management interventions for COPD patients [3] and none of these studies used computer-tailored technology. The results of this study explore the potential benefit of using computer-tailored technology to improve self-management related health behaviours of people at risk for COPD, in addition to those with COPD.

Individuals at risk for COPD are less likely to be offered a self-management intervention by a health care provider, but they can be reached relatively easily through the internet and health behaviour change can be promoted. We also expect that including people at risk for COPD and recruiting some of the participants online increases the likelihood of assuring adequate power.

The choice for a hybrid sample of participants recruited by two methods (online panel and general practice) was made after considering the results of the pilot study (unpublished observations). In the pilot study participants were recruited by a primary care practice nurse during a yearly consultation for routine monitoring of their chronic condition. This recruitment strategy resulted in an insufficient number of participants. Not all eligible patients were invited by the nurses and many patients declined participation. We concluded that it is not feasible to recruit 446 participants per arm using this approach. Therefore, we choose to recruit participants from an online panel and through general practices by sending an invitation letter to every eligible patient.

A possible threat to this study is that large numbers of participants might not use or discontinue to use the intervention, which is common in e-Health interventions [53]. If a large percentage of participants opt not to use the intervention, an intention to treat analysis could underestimate the intervention effect [54]. For this reason we will conduct a per-protocol analysis following an intention to treat analysis.

One of the strengths of this study is that we conduct a process evaluation to assess the implementation of the study protocol. A process evaluation enables researchers to explain study outcomes and helps understand why an intervention was effective or not [51]. As another advantage, recommendations can be formulated for further research in order to study specific aspects of the technology implementation.

## Abbreviations

CCQ: Clinical COPD Questionnaire; COPD: Chronic Obstructive Pulmonary Disease; GOLD: Global Initiative for Chronic Obstructive Lung Disease; IPAQ: International Physical Activity Questionnaire; ISO: International Organization for Standardization; MRC: Medical research council; RHSQ: The Respiratory Health Screening Questionnaire.

## Competing interests

Hein de Vries is scientific director of Vision2Health, a company that licenses evidence-based innovative computer-tailored health communication tools.

## Authors’ contributions

VV, HT, TvdW and HdV designed the original proposal and BW and ZN made significant contributions to the design. VV drafted the manuscript, while HT, TvdW, ZN, BW and HdV were involved in revising the manuscript critically. VV developed the intervention, based on earlier work of HdV. HdV contributed to the development of the feedback messages. HdV, TvdW and HT screened the intervention. All authors read and approved the final version of the manuscript.

## Pre-publication history

The pre-publication history for this paper can be accessed here:

http://www.biomedcentral.com/1471-2458/13/557/prepub
